# Comparison of clot lysis activity and biochemical properties of originator tenecteplase (Metalyse^®^) with those of an alleged biosimilar

**DOI:** 10.3389/fphar.2014.00007

**Published:** 2014-02-05

**Authors:** Werner Kliche, Ingo Krech, Martin C. Michel, Nishant V. Sangole, Sadhana Sathaye

**Affiliations:** ^1^Department of Biopharma Quality Control Germany, Boehringer Ingelheim Pharma GmbH & Co. KGBiberach, Germany; ^2^Department of Regional Medicine and Scientific Affairs, Boehringer Ingelheim Pharma GmbH & Co. KGIngelheim, Germany; ^3^Department of Pharmacology, Johannes Gutenberg UniversityMainz, Germany; ^4^Department of Medicine, Boehringer Ingelheim India Pvt. Ltd.Mumbai, India; ^5^Department of Pharmaceutical Science and Technology, Institute of Chemical TechnologyMumbai, India

**Keywords:** tenecteplase, biosimilar, clot lysis, glycosylation, impurity

## Abstract

The bioengineered tissue plasminogen activator tenecteplase is an important treatment modality of acute myocardial infarction recommended by international guidelines. Following introduction of originator tenecteplase (brand names Metalyse^®^ and TNKase^®^), a “biosimilar” tenecteplase became available for commercial use in India under the brand name Elaxim^®^ in the absence of Indian biosimilar guidelines which came into force from September 15th, 2012. Based on a report of biochemical and fibrinolytical differences between Metalyse and Elaxim, we have systematically compared them in a range of routine quality testing assays. As compared to Metalyse, Elaxim exhibited less clot lysis activity and contained less of the two-chain form of tenecteplase. Even upon full *in vitro* conversion to the two-chain form Elaxim exhibited less clot lysis activity. This was linked to differences in sialic acid content and glycosylation pattern with Elaxim exhibiting less bi- and more tetra-antennary glycosylation, leading to a different charge heterogeneity profile. Regarding purity, Elaxim contained more tenecteplase aggregates and, in contrast to Metalyse, considerable amounts of Chinese hamster ovary cell protein. Taken together these data demonstrate that Metalyse and Elaxim differ considerably in clot lysis activity and biochemical properties. These data question whether Elaxim indeed can be considered a “biosimilar” of Metalyse, i.e., whether and to which extent the clinical efficacy and safety properties of Metalyse can be extrapolated to Elaxim in the absence of comparative clinical data.

## INTRODUCTION

Acute myocardial infarction is a leading cause of heart failure and premature death (Kunadian and Gibson, 2012). In those who survive the acute phase, it may pose a financial catastrophe due to high out-of-pocket expenditures for acute cardiovascular care in countries lacking a comprehensive health insurance system, e.g., India (Mohanan et al., 2013). The acute treatment aims for early re-perfusion, preferably by primary percutaneous coronary intervention (Steg et al., 2012), but this may not be feasible due to a lack of early access to qualified facilities, e.g., in rural areas and in emerging market countries. The alternative treatment is fibrinolysis (Kunadian and Gibson, 2012). When performed within 6 h after symptom onset, fibrinolytic therapy prevents approximately 30 early deaths per 1000 treated patients with ST Elevation in Myocardial Infarction (Steg et al., 2012).

The serine protease tissue plasminogen activator (t-PA) is a physiologically occurring 527 amino acid glycoprotein and the main endogenous mediator of clot lysis. Its recombinant version rt-PA (alteplase) has become the standard fibrinolysis treatment in patients with acute myocardial infarction, pulmonary embolism, and acute ischemic stroke. The structure of t-PA is characterized by five regions with homology to other protein families, i.e., a finger, an epidermal growth factor-like, two kringle and a serine protease domain. In contrast to classic zymogens, the intact one-chain form of t-PA is already active in *in vitro* clot lysis assays, but full activity is obtained by cleavage between amino acids 275 and 276 to the two-chain form.

Tenecteplase has been modified from rt-PA (alteplase) by substitution of threonine 103 with asparagine, asparagine 117 with glutamine and a tetra-alanine substitution in position 296–299 (Davydov and Cheng, 2001). The mutation in position 103 created a new glycosylation site, hereby enlarging the molecule and increasing its half-life. The one in position 117 eliminated a high mannose-type side chain, which also contributed to prolonging the half-life. The one in positions 296–299 increased resistance to plasminogen activator inhibitor-1 (PAI-1). In comparison to rt-PA the combination of all three mutations resulted in an extended half-life (18 vs. 4 min), higher fibrin specificity (14-fold) and increased resistance towards PAI-1 (80-fold; Stewart et al., 2000; Davydov and Cheng, 2001). Accordingly, tenecteplase is the first and only therapeutic which can be used as a single dose bolus application during the early stages of an acute myocardial infarction (Llevadot et al., 2001; Melandri et al., 2009).

Similar to t-PA (Pohl et al., 1984), tenecteplase exhibits type I and II glycoforms; type I has three carbohydrate structures at asparagine residues 103, 184, and 448 (also termed glycosylation sites 1, 2, and 3 and located in the kringle 1, kringle 2, and protease domain, respectively), whereas type II lacks the carbohydrate at asparagine 184 (Parekh et al., 1989b). In contrast to alteplase, however, all carbohydrates in tenecteplase are complex oligosaccharides and no mannose structure is present, which prevents clearance by the hepatic mannose receptor that has been observed with alteplase (Tanswell et al., 2002). Of note, the specific glycosylation pattern depends on the host organism for expression of the genetically modified product and on other specific aspects of the manufacturing process (Schellekens, 2009); changes in glycosylation pattern can affect the ability of t-PA and tenecteplase to activate plasminogen (Parekh et al., 1989a).

Tenecteplase was introduced globally into medical practice in 1999–2000 under the brand names TNKase® (Roche/Genentech, South San Francisco, CA, USA) and Metalyse® (Boehringer Ingelheim Pharma GmbH & Co. KG, Ingelheim, Germany). Its efficacy and safety in the treatment of acute myocardial infarction has been documented in numerous randomized controlled clinical studies in which more than 15,000 patients received tenecteplase (Llevadot et al., 2001; Kunadian and Gibson, 2012). A “biosimilar” tenecteplase became available for commercial use in India under the brand name Elaxim® (Gennova Pharmaceuticals Ltd., Hinjewadi, Pune, India); it is also available in some other Asian countries. The only publicly available data on its efficacy and safety in the treatment of myocardial infarction come from an open label registry (Iyengar et al., 2011, 2013), which unfortunately lacks a clear definition and validation of the reported outcomes. The present study was designed to compare the clot lysis activity, purity, and glycosylation status of originator tenecteplase and its purported “biosimilar” variant.

## MATERIALS AND METHODS

All testing was performed by using International Conference on Harmonisation of Technical Requirements for Registration of Pharmaceuticals for Human Use validated methods according to standard operating procedures which are applied to the routine quality control testing of tenecteplase batches within Boehringer Ingelheim prior to release for market. An overview of method validation (intermediate precision) is given in **Table 1**. Testing was supplemented by state-of-the-art mass spectrometry analysis.

**Table 1:** Method validation for Boehringer Ingelheim Metalyse release tests.

Test method	Intermediate precision coefficient of variation (%)
Clot lysis activity assay	2
Chain composition by HP-SEC	1
Purity (monomer) by HP-SEC	1
Host cell proteins by CHOP ELISA	24
Type I/II by RP-HPLC	1

### CHEMICALS

Commercial Metalyse lot#22579 was used as comparator which is a representative lot for the highly consistent manufacturing process. In comparison two lots of commercial Elaxim (#140903 and #140904E04) were obtained from an Indian pharmacy. Thrombin, plasminogen, fibrinogen, and sialidase were obtained from Calbiochem (Merck, Darmstadt, Germany), respectively.

### *IN VITRO* CLOT LYSIS

Clot lysis was determined by an automated ACL TOP® hemostasis testing system (Instrumentation Laboratory, Bedford, MA, USA), in which the lysing effect is measured as a decrease of turbidity. Briefly, standard (5 concentrations, 400–1200 ng/ml) or multiple dilutions of tenecteplase were pipetted into sample cups (20 μl), thrombin solution (20 μl) was added and then the reaction was started by addition of a freshly mixed plasminogen/fibrinogen solution (200 μl) at 37°C. The measured lysis time was then plotted against tenecteplase concentration on a double-logarithmic scale. Enzyme activity was expressed as % of that of reference standard as assessed within the same experiment.

### CHAIN COMPOSITION

High-performance size-exclusion chromatography (HP-SEC) was used to quantify the one- and two-chain forms of tenecteplase. After reducing the disulfide bonds with dithiothreitol (DTT), the one-chain material was separated from the two-chain material based on its larger molecular weight and size. Analysis was monitored at 210 nm using a size-exclusion chromatography (SEC) column with a guard column (Tosoh Bioscience, Stuttgart, Germany) and sodium dodecyl sulfate (SDS)/sodium phosphate buffer as the mobile phase.

### PURITY ANALYSIS

#### HP-SEC

Tenecteplase aggregates, monomers as well as smaller cleavage products were separated by HP-SEC using a SEC column with a guard column (Tosoh Bioscience, Stuttgart, Germany). Analysis was monitored at 280 nm. An isopropanol/L-arginine/ammonium sulfate buffer was used as mobile phase.

#### SDS-PAGE (silver staining)

Tenecteplase samples were separated by SDS polyacrylamide gel electrophoresis (8–16% tris-glycine gels, constant current of 36 mA) under reducing conditions (sample buffer containing 160 mmol/l DTT). Thereafter, silver staining was performed by a modified Oakley method (Oakley et al., 1980).

#### Host cell proteins

Host cell proteins are process related impurities originating from the host cells used for manufacturing. The quantification of host cell protein, Chinese hamster ovary cell protein (CHOP), was performed by a product-specific enzyme-linked immunosorbent assay (ELISA) method on microtiter plates using a polyclonal CHOP antibody (goat anti-CHOP, prepared in-house). A polyclonal, horseradish peroxidase-coupled antibody was used with fluorescence detection of absorption at 490 nm and reference signal at 405 nm. Fluorescence signals were converted to units, with 1 U corresponding to 1 ng of CHOP standard.

### Type I/Type II DETERMINATION

Reverse-phase high-performance liquid chromatography (RP-HPLC) was used for the quantitation of the relative abundance of Type I and Type II forms. Type I has all 3 *N*-glycosylation sites occupied whereas the Type II form of tenecteplase has only two *N*-glycosylation sites occupied (kringle 1 and protease domain). Samples were treated with plasminogen to convert the tenecteplase completely into the two-chain form. The samples were then reduced with DTT to release the protease domain from the rest of the molecule composed of finger, epidermal growth factor-like, kringle 1, and kringle 2 domains, which were then separated by RP-HPLC. The method separates molecules on the basis of hydrophobicity. The parameters for the separation are a non-linear gradient with the buffer-system of trifluoroacetic acid and purified water as buffer A and trifluoroacetic acid and acetonitrile as buffer B. Protein absorption was monitored at 214 nm. For tenecteplase the more hydrophobic Type I elutes earlier than the Type II and the C-terminal portion of the tenecteplase (protease domain) elutes in the wash step of the acetonitrile gradient. Quantitation of Type I tenecteplase was achieved by determining the percent Type I peak area relative to the total Type I and Type II peak areas.

### GLYCOSYLATION, SIALYLATION, BRANCHING

In order to characterize and quantify complex N-linked oligosaccharides, assessed as the relative abundance of sialylated and non-sialylated bi-, tri- and tetra antennary complex N-linked oligosaccharides, AspN digested samples of Metalyse and Elaxim were analyzed by RP-HPLC coupled to electrospray ionization mass spectrometry. Briefly, samples were denatured using guanidine-HCl, reduced by DTT and alkylated with iodoacetic acid. Reduced and alkylated samples were subsequently digested using AspN (Roche, Mannheim, Germany). Resulting peptides were separated by RP-HPLC (C18-column) coupled with an ESI-Q-TOF premier mass spectrometer (Waters, Eschborn, Germany). MassLynx was used as data evaluation software. Separation of AspN peptides was achieved using an increasing acetonitrile elution gradient from 2% of mobile phase B (85% of acetonitrile in water, 0.1% of trifluoroacetic acid) to 45% of mobile phase B in 120 min. After the RP-HPLC run was completed and all peptides were eluted, the full-scan total ion current chromatogram was extracted for the corresponding high resolution masses of the glycopeptides as selective ion chromatogram for each of the three N-linked glycosylation sites [glycopeptide 1 (kringle 1): DQGI-SYRGNWSTAESGAECTNWQSSALAQKPYSGRRP; glycopeptide 2 (kringle 2): DCYFGNGSAYRGTHSLTESGASCLPWNSMILIG-KVYTAQNPSAQALGLGKHNYCRNP; glycopeptide 3 (protease domain): DWTECELSGYGKHEALSPFYSERLKEAHVRLYPSSR-CTSQHLLNRTV (the glycosylation site is underlined)]. For each glycosylation site the summarized signal intensities of the related mass/charges (m/z) of the selected ion chromatograms of the glycopeptides were integrated and summarized to 100% and then the ratio was calculated for sialylated and non-sialylated bi-, tri- and tetra-antennary complex N-linked oligosaccharides. Based on these data the degree of sialylation for each glycosylation site was calculated as follows: sialylation = [Σ mono-sialylated structures (%) + 2 x Σ bi-sialylated structures (%) + 3 x Σ tri-sialylated structures (%) + 4 x Σ tetra-sialylated structures (%)]. As glycopeptide 2 is not fully occupied, a correction for site occupancy was performed. Total sialylation was calculated as the sum of the three individual sialylations and expressed as either mol/mol or % values relative to Metalyse® reference standard. The degree of free terminal hexoses was determined after removal of sialic acid residues using sialidase, analogously as described above for total sialylation.

### ISOELECTRIC FOCUSING

To determine the charge heterogeneity of tenecteplase as a result of different branching and sialylation, isoelectric focusing was performed using a Phast system and PhastGels IEF 5–8 (GE Healthcare, Freiburg, Germany). PhastGels IEF 5–8 were equilibrated and run according to the manufacturer’s instructions. After focusing, the gels were stained using Coomassie G250 by a modified method according to Neuhoff et al. (1985).

### STATISTICAL ANALYSIS

Our study is based on highly standardized and well validated quality control release assays. Across all quantitative assays both tested Elaxim batches consistently differed from the representative Metalyse batch to a degree which considerably exceeds the precision of the respective method (**Table 1**). Therefore, experiments were not repeated multiple times, and hence statistical analysis was not performed.

## RESULTS

### *IN VITRO* CLOT LYSIS

**FIGURE 1:**
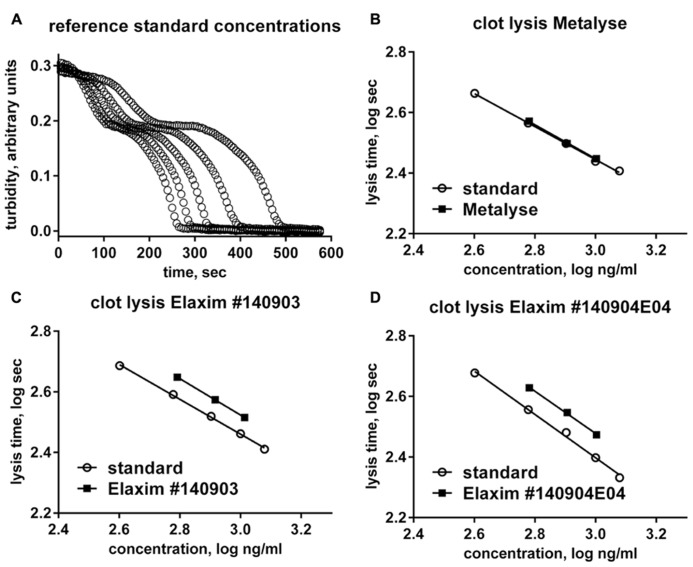
***In vitro* clot lysis by one batch of Metalyse and two batches of Elaxim. (A)** Raw data for the time course of turbidity change induced by 400, 600, 800, 1000, and 1200 ng/ml tenecteplase reference standard. **(B–D)** Double-logarithmic plot of clot lysis time vs. concentration of test compound in comparison to matched tenecteplase reference standard. Based on these data the calculated *in vitro* clot lysis activity for Metalyse was 97% as compared to 77 and 76% for the two batches of Elaxim.

For each tested concentration, the two batches of Elaxim required a longer time to cause lysis than the tenecteplase reference standard, whereas lysis time of Metalyse matched values from the reference standard (**Figure 1**). From these findings tenecteplase activities of 77% and 76% for Elaxim #140903 and #140904E04, respectively, were determined as compared to 97% for Metalyse (**Figure 1**). A lower biological activity of the two lots of Elaxim (72–78%) was confirmed in two other assays, i.e., the activity assay performed using an alternative quality control assay and the clot lysis activity method described in the European Pharmacopoeia monograph on alteplase, where Metalyse exhibited activities of 99–100% (data not shown).

### CHAIN COMPOSITION AND *IN VITRO* CHAIN CONVERSION

**FIGURE 2:**
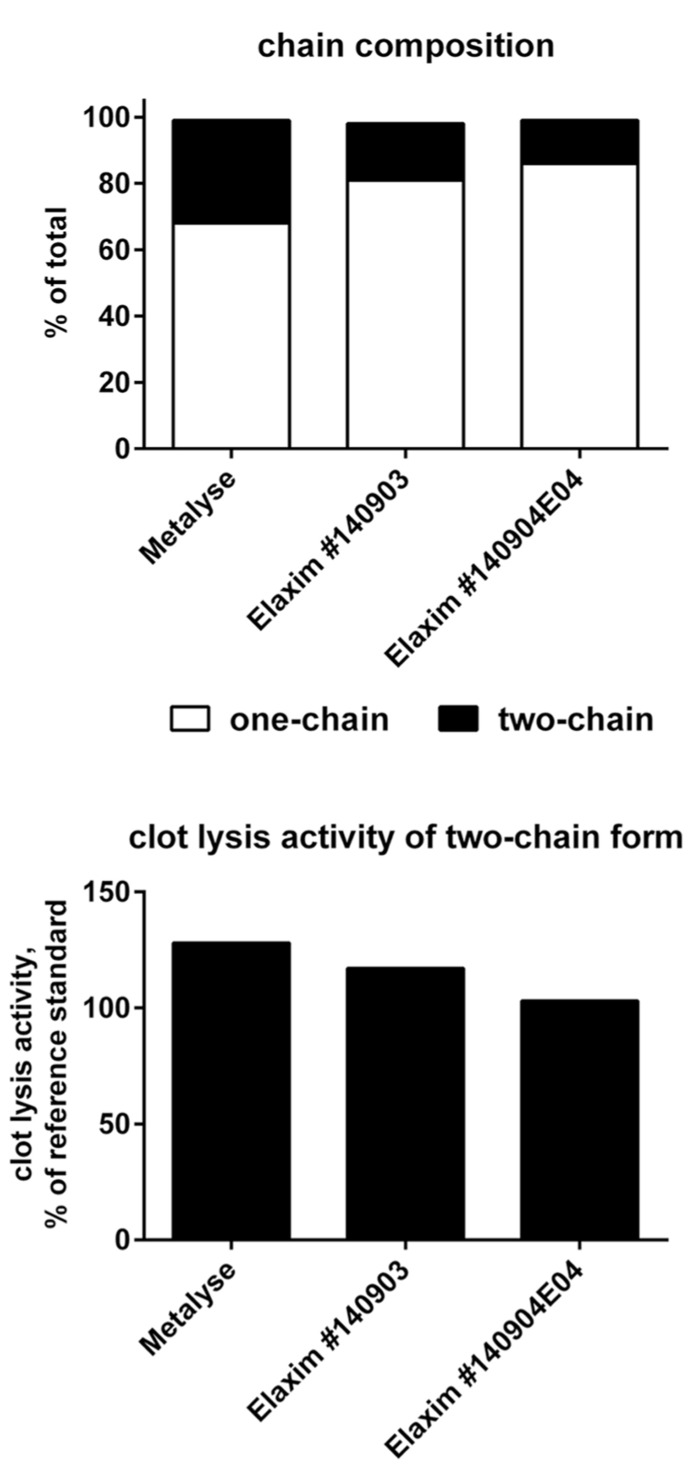
**Relative abundance of one- and two-chain form (upper panel) and *in vitro* clot lysis activity of Elaxim and Metalyse enzymatically converted to two-chain form (lower panel)**.

The two-chain form is the most active form of tenecteplase. In confirmation of previous studies (Jiang et al., 2010), the relative abundance of the two-chain form in the two Elaxim batches was only about half that of Metalyse (**Figure 2**). After *in vitro* enzymatic conversion of tenecteplase yielding 95–97% two-chain form for both tested batches, clot lysis activity was determined and found to be lower for the two Elaxim batches as compared to Metalyse (**Figure 2**).

### PURITY ANALYSIS

**FIGURE 3:**
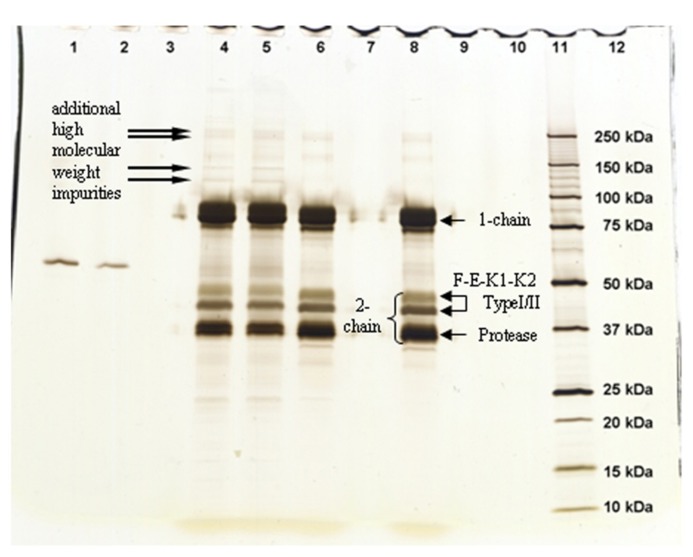
**Silver staining of gel from SDS electrophoresis under reducing conditions to detect tenecteplase aggregates and fragments.** Lanes: 1–2 bovine serum albumin staining control; 4 Elaxim #140903; 5 Elaxim #140904E04; 6 Metalyse; 8 reference standard; 11 molecular weight markers. The right-facing arrows point to additional high molecular weight impurities.

Three types of experiments were performed for purity analysis. Firstly, in the SEC assay, 98.4% of Metalyse was found as monomer, 1.0% as aggregates and 0.6% as fragments. In contrast, both Elaxim lots #140903 and #140904E04 contained only 96.4% monomers but 2.9% aggregates and 0.4% fragments. Secondly, in SDS polyacrylamide gel electrophoresis experiments under reducing conditions and with silver staining detection, Elaxim lots #140903 and #140904E04 exhibited high molecular weight impurities (**Figure 3**). Thirdly, in the ELISA testing for presence of CHOP, Elaxim batches #140903 and #140904E04 yielded 6590 and 6990 U, respectively, whereas Metalyse yielded < 15 U (the lower limit of quantification).

### GLYCOSYLATION, SIALYLATION, AND BRANCHING

In RP-HPLC experiments, the relative abundance of type I tenecteplase was 42% for Metalyse as compared to 37% and 34% for Elaxim batches #140903 and #140904E04, respectively.

**FIGURE 4:**
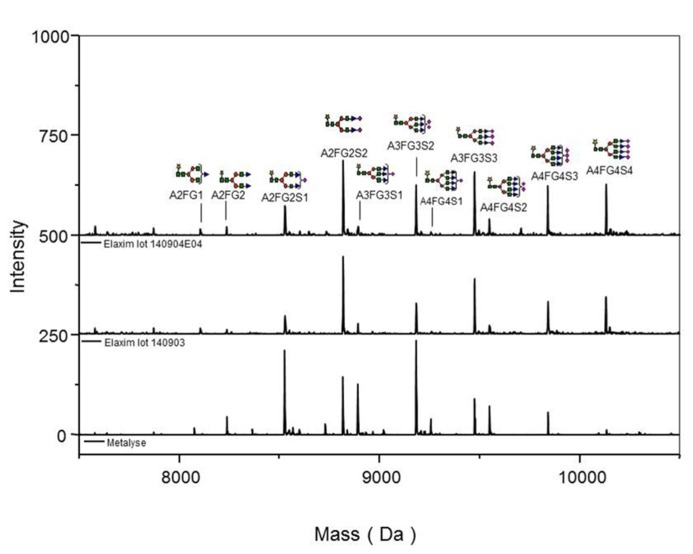
**Deconvoluted spectra of glycopeptide 2 (kringle 2) acquired on an ESI-Q-TOF instrument, for Metalyse® lot #22579 and Elaxim lots #140903 and #140904E04**.

**FIGURE 5:**
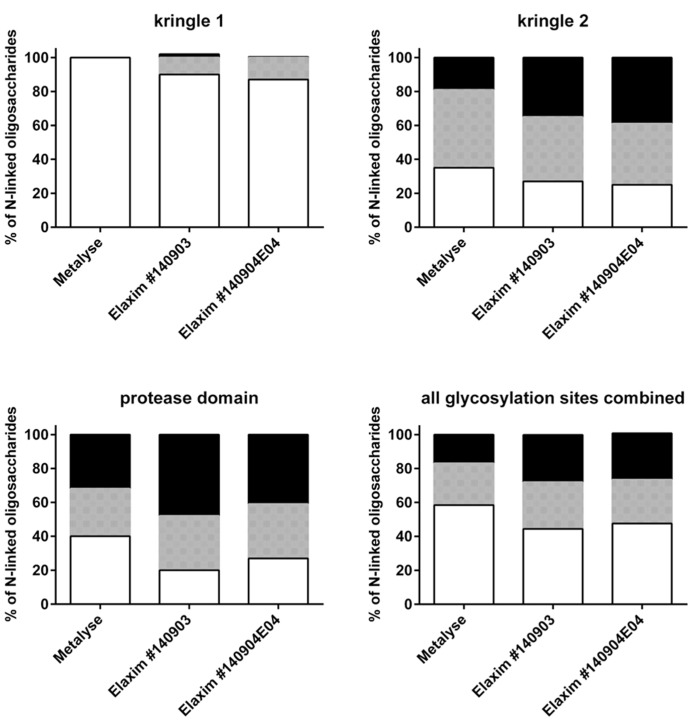
**Relative abundance of bi-, tri- and tetra-antennary N-linked oligosaccharides (indicated as white, grey, and black bars, respectively) at glycosylation sites in kringle 1, kringle 2, and protease domain and in the overall glycosylation sites**.

Glycosylation was analyzed by mass spectrometry (RP-HPLC-ESI-MS); **Figure 4** shows examples of the deconvoluted spectra for Metalyse and Elaxim lots #140903 and #140904E04 for the glycopeptide 2 (kringle 2). Both Elaxim lots exhibited a lower percentage of bi-non-sialylated N-linked oligosaccharides at glycosylation site 1 as compared to Metalyse, whereas tri- and tetra-sialylated glycosylation was observed with both Elaxim lots but not with Metalyse (**Figure 5**), indicating a higher degree of tri- and tetra-antennary branching at glycosylation site 1 in kringle 1. At glycosylation sites 2 and 3, the Elaxim batches also inhibited less bi- and more tetra-antennary N-linked oligosaccharides than Metalyse (**Figure 5**). Accordingly, combined data for all three glycosylation sites indicated reduced bi- and increased tetra-antennary glycosylation in Elaxim as compared to Metalyse (**Figure 5**). To corroborate these findings the total sialylation and free terminal hexose content after removal of sialic acid residues of the tested tenecteplase preparations was determined. Metalyse, Elaxim batch #140903, and Elaxim batch #140904E04 had 3.6, 5.0, and 5.0 mol sialic acid/mol tenecteplase, respectively, corresponding to 98, 130, and 136% of reference standard, respectively. Free terminal hexoses after removal of sialic acid measurement resulted in 5.5, 6.2, and 6.3 mol free hexose per mol tenecteplase for Metalyse and Elaxim lots #140903 and #140904E04, respectively. There is a fourth O-linked fucose glycosylation site at position threonine 61 in the EGF domain, for which comparable results (fully glycosylated) was found for both Metalyse and the two Elaxim batches (data not shown), which is in line with published data (Jiang et al., 2010).

**FIGURE 6:**
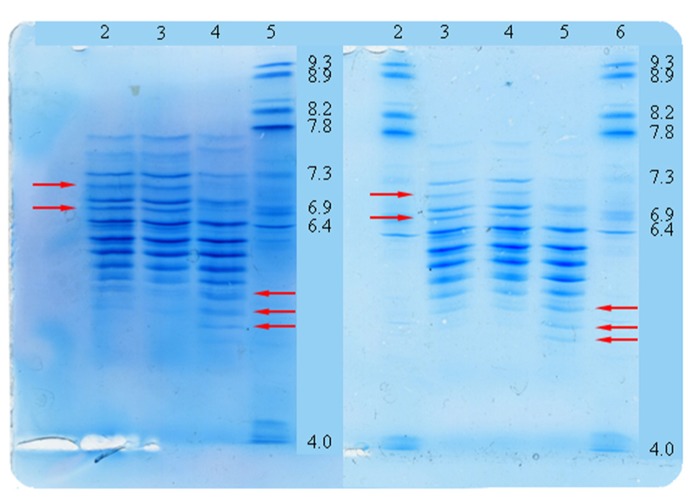
**Tenecteplase charge heterogeneity as detected by isoelectric focusing.** Lanes left panel: 2 reference standard; 3 Metalyse; 4 Elaxim #140904E04; 5 pl-marker. Lanes right panel: 2 pl-marker; 3 reference standard; 4 Metalyse; 5 Elaxim #140903; 6 pl-marker. Arrows indicate shift of bands to the acidic region as compared to reference standards.

Finally isoelectric focusing was used to determine the charge heterogeneity. This demonstrated a shift to the acidic region for both Elaxim lots yielding an overall pattern distinct from that of Metalyse (**Figure 6**).

## DISCUSSION

A pharmaceutical product is considered a biosimilar if it is highly similar to an already approved biological product, notwithstanding minor differences in clinically inactive components (Schellekens, 2009). If similarity including purity is high, it is assumed that the efficacy and safety of the biosimilar can be extrapolated from the originator drug. Based on this assumption, biosimilars often receive regulatory approval based on more limited experimental and clinical data packages than the originator biological agent. However, in contrast to traditional generics and based on their more complex three-dimensional structures, biosimilars are often more fragile physically and chemically, and have more stringent formulation and storage requirements than small molecule-based generic drugs (Kuhlmann and Marre, 2010).

Biosimilars are attractive from a public health perspective as they typically are less expensive and hence can make treatment more affordable. However, in the interest of safe and efficacious treatment this consideration only applies if the biosimilar does not exhibit clinically relevant differences to the originator product. The verification of similarity between the innovator biopharmaceutical and the biosimilar is a major challenge, as protein products generally exhibit microheterogeneities, which may affect biological activity. Therefore, the present study was designed to compare the “biosimilar” variant Elaxim to the innovator tenecteplase Metalyse in terms of activity and purity status in a range of quality control assays, as well as glycosylation status analyzed by mass spectrometry. Of note, all of the quantitative differences between representative lots of Metalyse and Elaxim reported here markedly exceed the variance of the test methods being applied (**Table 1**).

Previous studies have demonstrated that Metalyse and Elaxim do not differ in their primary amino acid sequence or the 17 intra-molecular disulfide linkages (Jiang et al., 2010). The glycosylation occupancy at asparagine 184, differentiating Type I and Type II tenecteplase, was reported to be approximately 60% for Metalyse as compared to 25% for Elaxim (Jiang et al., 2010). As changes in glycosylation affect the ability of t-PA and tenecteplase to activate plasminogen (Parekh et al., 1989a), we first compared the functional relevance of such glycosylation differences on clot lysis activity. Unlike the expectation that a lower degree of glycosylation at asparagine 184 is linked with higher clot lysis activity, the clot lysis activity of Elaxim was only 72–78% of the reference standard as compared to 99–100% for Metalyse; this lower biological activity of Elaxim was consistent across two batches of Elaxim and three distinct assays. Considering the intermediate precision of the clot lysis activity assay (**Table 1**), these data demonstrate that the biological activity of Elaxim differs from that of Metalyse. Our subsequent experiments were designed to explore the reasons for this difference.

As the two-chain form is the most active form of tenecteplase, we have compared the one- vs. two-chain content of Metalyse and Elaxim. In confirmation of data from other investigators (Jiang et al., 2010), we found a lower two-chain content in two Elaxim batches as compared with Metalyse. Jiang et al. (2010) had argued that “any single-chain form should convert into the two-chain forms on contact with plasma” and hence such difference should contribute little to differences in biological activity. Rather they proposed that consistency of the % of two-chain production should be a gauge of the manufacturer’s reproducibility. Our experiments extend this line of thinking by demonstrating that even upon full *in vitro* conversion to the two-chain form, two batches of Elaxim consistently exhibited less clot lysis activity than Metalyse, indicating possible molecule-intrinsic differences (see next paragraph).

Our data with tenecteplase fully converted to the two-chain form indicate that Elaxim may exhibit intrinsically less clot lysis activity than Metalyse. As both tenecteplase products do not differ in their primary amino acid sequence or the 17 intra-molecular disulfide linkages (Jiang et al., 2010), a difference in glycosylation pattern of the polypeptide may contribute. Of note, glycosylation patterns are not only dependent on primary amino acid sequence but also on the particular cell type used for expression of the recombinant polypeptide in the manufacturing process. t-PA contains three potential N-glycosylation sites, of which either three (Type I) or two (Type II) are occupied (Pohl et al., 1984). In our HPLC experiments, the relative abundance of Type I was 42% for Metalyse as compared to 34–37% for the two Elaxim batches. As Type II tenecteplase is considered to be more active (Parekh et al., 1989a), the lower percentage of Type I would suggest that Elaxim has greater clot lysis activity, but the opposite was found. This could be linked to its greater single-chain content and/or to difference in glycosylation. Moreover, oxidation of t-PA has been proposed to reduce fibrin binding and clot lysis activity (Stief et al., 1991), and the relative percentage of oxidation at methionine 207, 445, and 490 was higher with Elaxim than with Metalyse (Jiang et al., 2010).

This prompted us to explore the glycosylation patterns of Elaxim and Metalyse in more detail. At each of the three investigated N-linked glycosylation sites, i.e., kringle 1, kringle 2, and protease domain, the two Elaxim batches exhibited a different relative abundance of bi-, tri- and tetra-antennary N-linked oligosaccharides, yielding an overall glycosylation pattern with less bi- and more tetra-antennary glycosylation. This was confirmed by quantification of free terminal hexoses after removal of sialic acid. Based on the known relationship between glycosylation site occupancy and biological activity in the clot lysis assay (Jiang et al., 2010), these differences in glycosylation pattern would suggest that the higher branching (more tetra-antennary N-linked oligosaccharides), as well as the higher total sialylation may contribute to a decreased clot lysis activity of Elaxim.

To explain lower clot lysis activity of Elaxim, found to be smaller even after full conversion to the two-chain form, three types of experiments were performed to test whether possibly contributing impurities also are present. These demonstrated a higher fraction of fragments in two Elaxim batches than in Metalyse. Moreover, electrophoresis with silver staining detected higher molecular weight impurities in Elaxim which were absent in Metalyse. Finally, an ELISA detected substantial amounts of CHOP in both Elaxim batches which were below the quantification limit in Metalyse. Thus, three direct assays demonstrated that Elaxim exhibited a greater degree of impurities than Metalyse. These findings substantiate the proposal by Jiang et al. (2010) with regard to manufacturing reproducibility of Elaxim.

In summary, previous (Jiang et al., 2010) and present data demonstrate that Elaxim has less clot lysis activity than Metalyse. This was linked to a lower abundance of the two-chain form, a quantitatively different glycosylation pattern leading to a different charge heterogeneity profile and a greater methionine oxidation. Moreover, the tested Elaxim batches exhibited a greater percentage of tenecteplase aggregates as well as contamination with host cell protein. For some of these parameters there was also inconsistency between the tested Elaxim batches. It is possible that these biochemical differences may also contribute to differential storage requirements, i.e., according to the respective prescribing information Elaxim needs to be stored under refrigeration at 2–8°C whereas an unopened vial of Metalyse can be stored at up to 30°C. These findings suggest that Elaxim may not be a “biosimilar” to Metalyse according to internationally accepted standards. Hence, the reported differences between the two types of tenecteplase may translate into differences in efficacy and safety and it does not appear justified to extrapolate and apply the extensive data from clinical Metalyse studies (Llevadot et al., 2001; Melandri et al., 2009) to Elaxim. In view of differences in the structure, impurities, and related fibrinolytic activity as compared to Metalyse, extensive dedicated non-clinical and randomized controlled clinical studies are required to characterize the efficacy and safety profile of Elaxim in order to unequivocally define its place in the treatment of acute myocardial infarction.

## AUTHOR CONTRIBUTIONS

Werner Kliche was involved in design of the study, acquisition of data and supervision of data acquisition, revised the manuscript for important intellectual content, approved the final version and agrees to be accountable for all aspects of the work. Ingo Krech was involved in design of the study, acquisition of data and supervision of data acquisition, revised the manuscript for important intellectual content, approved the final version and agrees to be accountable for all aspects of the work. Martin C. Michel was involved in design of the study, contributed to the primary draft of the manuscript, revised the manuscript for important intellectual content, approved the final version and agrees to be accountable for all aspects of the work. Nishant V. Sangole contributed to the primary draft of the manuscript, revised the manuscript for important intellectual content, approved the final version and agrees to be accountable for all aspects of the work. Sadhana Sathaye was involved in design of the study, contributed to the primary draft of the manuscript revised the manuscript for important intellectual content, approved the final version and agrees to be accountable for all aspects of the work.

## Conflict of Interest Statement

Sadhana Sathaye reports no conflict of interest.All other authors are employees of the Boehringer Ingelheim family of companies.
